# Supplementary material for: Long-Term Maintenance of Sinus Rhythm Is Associated with Favorable Echocardiographic Remodeling and Improved Clinical Outcomes after Transcatheter Aortic Valve Replacement
Source: J Clin Med. 2022 Feb 28;11(5):1330. doi: 10.3390/jcm11051330 (PMC8911407; doi:10.3390/jcm11051330)
Supplement: Supplementary file 1 [file jcm-11-01330-s001.zip › jcm-1588205-supplementary.pdf]

Table S1. The primary and the secondary endpoints according to rhythm status in different types of periprocedural AF

	**Pre-existing AF**			**NOAF**		
	**SRM** (***N*** **= 19)**	**AF sustained** (***N*** **= 33)**	***p***	**SRM** (***N*** **= 24)**	**AF sustained** (***N*** **= 11)**	***p***
Death, stroke or rehospitalization	3 (15.8%)	8 (24.2%)	0.312	2 (8.3%)	8 (72.7%)	0.002
All cause death	3 (15.8%)	3 (9.1%)	0.610	1 (4.2%)	4 (36.4%)	0.049
Cardiovascular death	1 (5.3%)	2 (6.1%)	0.827	1 (4.2%)	3 (27.3%)	0.104
HF rehospitalization	0	6 (18.2%)	0.127	0	4 (36.4%)	0.010
Stroke	0	0	NS	1 (4.2%)	0	NS
Major bleeding	1 (5.3%)	4 (12.1%)	0.383	1 (4.2%)	1 (9.1%)	0.419

p-values were calculated using univariate Cox-regression analysis. $p < 0.05$ indicates statistical significance.

AF = atrial fibrillation; NOAF = new onset atrial fibrillation; SRM = sinus rhythm maintained; HF = heart failure.

Table S2. Predictors for long-term sinus rhythm maintenance in patients with periprocedural AF.

Variable	Crude HR	95% CI	*p*	Adjusted HR	95% CI	*p*
Age	1.021	0.953–1.094	0.582	1.013	0.890–1.137	0.931
Male sex	1.385	0.592–3.224	0.457	2.118	0.475–9.526	0.333
LVEF < 50%	0.913	0.341–2.436	0.849	2.212	0.432–11.455	0.344
BMI	1.012	0.918–1.127	0.891			
Balloon-expandable device	0.301	0.126–0.734	0.008	0.964	0.242–3.905	0.952
Hypertension	0.860	0.321–2.292	0.755			
Diabetes	0.785	0.322–1.937	0.594			
Stroke	0.895	0.324–2.573	0.827			
Prior MI	0.866	0.261–2.790	0.798			
Anti-arrhythmic drug	1.410	0.584–3.462	0.449	1.107	0.285–4.284	0.892
CKD	0.558	0.226–1.494	0.236	1.451	0.292–7.283	0.648
CHA_2DS_2-Vasc score	0.981	0.750–1.273	0.861			
Paroxysmal type	28.576	7.533–108.429	<0.001	24.913	4.116–150.882	<0.001
LAVI < 46 ml/m^2	14.874	3.861–57.252	<0.001	13.320	2.281–77.694	0.004

HR and p-value were calculated using univariate and multivariate logistic regression analysis. P <0.05 indicates statistical significance. AF = atrial fibrillation; HR = hazard ratio; CI = confidence interval; LVEF = left ventricular ejection fraction; BMI = body mass index; MI = myocardial infarction; CKD = chronic kidney disease; LAVI = left atrial volume index
